# Supplementary material for: Cutaneous Leishmania mexicana infections in the United States: defining strains through endemic human pediatric cases in northern Texas
Source: mSphere. 2024 Feb 29;9(3):e00814-23. doi: 10.1128/msphere.00814-23 (PMC10964424; doi:10.1128/msphere.00814-23)
Supplement: Supplemental materials — Supplemental text, figure, and table. [file msphere.00814-23-s0001.pdf]

SUPPLEMENTAL MATERIAL

Supplemental Methods, Results and Discussion:

Additional specific details regarding genetic sequencing of the clinical isolates from these patients are described below. Of interest, this analysis can be done after sampling lesions with a flocked nylon swab, which provides a less invasive means of diagnosis in children. All sequences described in this manuscript have been submitted to NCBI (GeneBank).

ITS2: We performed sequence analysis on the rRNA-internal transcribed spacer 2 (ITS2) region, which shows a previously reported Texas-specific polymorphism, C647 and C649, on both Tx2 and Tx3. For this analysis, FJ948434 was used as a reference strain. However, alignments of the FJ948434 ITS2 sequence with 25 publicly available non-US *L. mexicana* ITS2 sequences were identical. Isolates' ITS2 sequences are shown in the **Supplemental Figure**, and primer sequences are contained in the **Supplemental Table**.

Metabolic enzymes: For the three metabolic enzymes sequenced, *Leishmania mexicana* MHOM/GT/2001/U1103 (Guatemala) was used as a reference strain, since it is the *L. mexicana* strain with the most widely available genomic sequence *(1)*.

MDH: SNP analysis in the CDS of the MDH gene showed a total of 15 SNPs in Tx2. We identified nine base transitions: 185 (T→C), 223 (A→G), 226 (C→T), 300 (T→C), 332 (T→C), 515 (A→G), 523 (A→G), 846 (C→T), 868 (A→G), 869 (T→C), and five base

transversions, 184 (T→A), 292 (A→C), 378 (T→G), 922 (A→T), 946 (T→A). Tx3 had two SNPs, a base transition at 523 (A→G) and a transversion at 292 (A→C). We note that SNPs 184 (T→A), 185 (T→C), 223 (A→G), 226 (C→T), 515 (A→G), 523 (A→G), 868 (A→G), 869 (T→C) were nonsynonymous and resulted in amino acid changes (**Figure 2, Supplemental Table**). Of particular interest, MDH in Tx2 was prematurely truncated (by 9 aa at the C terminus) due to transversion 922 (A→T). However, this truncation would not be expected to affect its enzymatic function *(2)*.

PGD: Similarly, we identified 2 SNPs on coding region of PGD gene of Tx2, transversion at 729 (G→T) and single transition 990 (A→G). Tx3 had a single base transition at 990 (A→G).

MPI: The MPI gene for the Tx3 isolate had 3 base transitions, 592 (A→G), 612 (T→C), 661 (T→C). On the other hand, for Tx2, the MPI gene had 20 SNPs: transitions 252 (G→A), 417 (C→T), 501 (A→G), 504 (C→T), 529 (A→G), 592 (A→G), 612 (T→C), 661 (T→C), 693 (A→G), 711 (T→C), 720 (G→A), 1148 (A→G), 1182 (G→A) and transversions: 483 (A→T), 493 (A→T), 545 (C→G), 605 (C→A), 663 (C→ G), 870 (C→A), 1032 (C→G). SNPs 529 (A→G), 545 (C→G), 592 (A→G), 605 (C→A), 661 (T→C), 663 (C→G), 1148 (A→G) were nonsynonymous.

Supplemental Figure

```
FJ948434.1    ATACACACATGTGCACTCTCCTTTGTGTGGGTGCGCGCGTGGAAAAACTCCTCTCTGGTG    660
Tx2.ITS2      ATACACACATGTGCACTCTCCTTTGTGTGGGTGCGCGCGTGGAAAACCCCCTCTCTGGTG    224
Tx3.IT2       ATACACACATGTGCACTCTCCTTTGTGTGGGTGCGCGVGTGGAAAACCCCCTCTCTGGTG    224
              ********************************************** * ***********
```

Supplemental Figure. Sequences of the relevant ITS2 region in a representative non-US reference strain (FJ948434.1), compared to Tx2 and Tx3.

Supplemental Table

Gene	Accession number	Amplicon size (bp)	CDS length (bp)	Location	Primer sequence
ITS2		Variable	N/A	18S ribosome	ITS2.F: GCATGCCATATTCTCAGTGTC<br>ITS2.R: GGCCAACGCGAAGTTGAATTC<br>ITS2.R1: GAATTCTCGTTTTGGTTTTTTG
Glucose-6-phosphate dehydrogenase (*g6pdh)*	XM_003879281.1	1700	1440	Chr 34	ExtF: TTACTGGCGCTTAGTCCCATCATA<br>ExtR: TAAGAGCGGAATCGGGTGC<br>IntF: GCAGGCAACTATCTGTCAGAGAA<br>IntR: ATGTTGAGCGAGGGCGCTG
Malate dehydrogenase (*mdh*)	XM_003878549.1	1166	951	Chr 33	ExtF: TGCCGTACAATCCATCTCGC<br>ExtR: AACGACGCCGCATCATTGTG<br>IntF: ATCATCAGCAACCCTGTCAACAGC<br>IntR: TAACGTCGTACGGGCTTGC
Mannose phosphate isomerase (*mpi*)	XM_003879281	1471	1266	Chr 31	ExtF: TCTGGGTTGTGTAGTAGCGTC<br>ExtR: AATTCGCCTGCCACTGTG<br>IntF: TCCAGAGCTGGCTGAGCTTGTAG<br>IntR: CATACACGGTCGTCATCATAGTC<br>SeqF1: CTTTGAGGCACTCTGCTGC<br>SeqF2: GACTATGATGACGACCGTGTATG<br>SeqR: GTCGTATCGTTCACCTTGGCAG<br>SeqR1: CGTCAATGTTGAAGGAAACTCGGT

Supplemental Table. Genes and primer sequences used for MLSA analysis.

SUPPLEMENTAL REFERENCES

1. Rogers MB, Hilley JD, Dickens NJ, Wilkes J, Bates PA, Depledge DP, et al. Chromosome and gene copy number variation allow major structural change between species and strains of Leishmania. Genome Res. 2011 Dec;21(12):2129-42.
2. Leroux A, Fleming-Canepa X, Aranda A, Maugeri D, Cazzulo JJ, Sánchez MA, and Nowicki C. Functional characterization and subcellular localization of the three malate dehydrogenase isozymes in Leishmania spp. Mol Biochem Parasitol. 2006: 149(1):74-85.
